# GGA1 participates in spermatogenesis in mice under stress

**DOI:** 10.7717/peerj.15673

**Published:** 2023-08-03

**Authors:** Haoyun Jiao, Yinghong Chen, Tingting Han, Qiyu Pan, Fei Gao, Guoping Li

**Affiliations:** 1 The Key Laboratory of Geriatrics, Beijing Institute of Geriatrics, Institute of Geriatric Medicine, Chinese Academy of Medical Sciences, Beijing Hospital/National Center of Gerontology of National Health Commission, Beijing, P.R. China; 2 State Key Laboratory of Stem Cell and Reproductive Biology, Institute of Zoology, Chinese Academy of Sciences, Beijing, P.R. China; 3 University of Chinese Academy of Sciences, Beijing, P.R. China; 4 Guangzhou Women and Children’s Medical Center, Guangzhou Medical University, Guangzhou, P.R. China; 5 Beijing Institute for Stem Cell and Regenerative Medicine, Beijing, China; 6 Institute for Stem Cell and Regeneration, Chinese Academy of Sciences, Beijing, China

**Keywords:** *Gga1*, BPA, Sperm count, Sperm morphology, Spermatogenesis

## Abstract

**Background:**

Infertility is recognized as a common and worrisome problem of human reproduction worldwide. Based on previous studies, male factors account for about half of all infertility cases. Exposure to environmental toxicants is an important contributor to male infertility. Bisphenol A (BPA) is the most prominent toxic environmental contaminant worldwide affecting the male reproductive system. BPA can impair the function of the Golgi apparatus which is important in spermatogenesis. GGA1 is known as Golgi-localized, gamma adaptin ear-containing, ARF-binding protein 1. Previously, it has been shown that GGA1 is associated with spermatogenesis in *Drosophila*, however, its function in mammalian spermatogenesis remains unclear.

**Methods:**

*Gga1* knockout mice were generated using the CRISPR/Cas9 system. *Gga1*^-/-^ male mice and wild-type littermates received intraperitoneal (i.p.) injections of BPA (40 µg/kg) once daily for 2 weeks. Histological and immunofluorescence staining were performed to analyze the phenotypes of these mice.

**Results:**

Male mice lacking *Gga1* had normal fertility without any obvious defects in spermatogenesis, sperm count and sperm morphology. *Gga1* ablation led to infertility in male mice exposed to BPA, along with a significant reduction in sperm count, sperm motility and the percentage of normal sperm. Histological analysis of the seminiferous epithelium showed that spermatogenesis was severely disorganized, while apoptotic germ cells were significantly increased in the *Gga1* null mice exposed to BPA. Our findings suggest that *Gga1* protects spermatogenesis against damage induced by environmental pollutants.

## Introduction

Infertility is defined as the failure of a couple to achieve a pregnancy after >12 months of regular, unprotected sexual intercourse by the World Health Organization (WHO) (Vander Borght & Wyns, 2018). Infertility is a global health issue affecting about 15% couples of reproductive age worldwide (Li, Yang & Wu, 2022). Statistically, infertility caused by male factors accounts for about 50% of infertility cases overall (Krausz, 2011). Many environmental factors (*e.g.*, BPA, dioxin, cadmium, diazinon, smoking) have been identified as risk factors for male infertility (Adamkovicova et al., 2016; Mínguez-Alarcón, Chavarro & Gaskins, 2018; Mohammadi et al., 2019; Park et al., 2020). BPA, the main chemical monomer of epoxy resins and polycarbonate plastics, is often used to make food containers, water bottles, children’s toys, and medical devices (Hu et al., 2017). Due to its widespread use, 92.6% of Americans were tested positive for BPA with the 95th percentile concentration up to 15.9 µg/L (Calafat et al., 2008). A large number of studies have demonstrated that BPA exposure can exert adverse effects on male reproductive function (Liang, Dai & Sun, 2020). In recent years, numerous clinical studies have confirmed that exposure to BPA results in reduced male fertility, decreased testis weight, inhibited spermatogenesis, and disturbed blood-testis barrier (BTB) (Chianese et al., 2018a; Chianese et al., 2018b; Chioccarelli et al., 2020; Li et al., 2021; Santoro et al., 2019). Nowadays, animal experiments have shown that the symptoms of sterile male mice are similar to those of human male infertility caused by BPA, such as a significant decrease in sperm count and motility, and obvious changes in testicular histology (Salian, Doshi & Vanage, 2009). BPA, as an endocrine-disrupting chemical (EDC), has a similar core structure to natural 17 *β*-estradiol (E2) and affects the endocrine system by binding to estrogen receptors (Wang et al., 2016). Besides that, BPA impairs the normal function of the organelles such as endoplasmic reticulum (ER) and Golgi apparatus (Pan et al., 2021). However, the mechanism of impaired spermatogenesis caused by BPA exposure remains to be further explored.

The GGAs are a family of monomeric clathrin adaptor proteins and are involved in the transport of cargo proteins between the trans-Golgi network (TGN) and endosomes (Bonifacino, 2004). GGAs are evolutionarily conserved from yeast to mammals. However, there is only a single GGA ortholog in *Drosophila melanogaster* (Hirst & Carmichael, 2011). All GGAs have a similar structure containing four functional regions, from the N-terminal to the C-terminal: VHS (Vps27/Hrs/STAM) domain, GAT (GGA and Tom1) domain, hinge region, and GAE (Gamma-adaptin ear) domain (Govero et al., 2012). The VHS domain binds to the acidic-cluster dileucine (AC-LL) motifs in the cytoplasmic tail of cargo molecules and promotes the incorporation of these molecules into forming clathrin-coated carriers which deliver them to the endosome. The GAT domain binds to ARF-GTP and PI4P, which allows GGAs to be recruited from the cytosol onto the TGN (Boman et al., 2000). In addition, the GAE domain binds to accessory proteins.

The main function of GGAs is to classify many proteins into clathrin-coated vesicles, such as mannose 6-phosphate receptor (MPR), Sortilin, sorLA, Stabilin-1, TrkA, LDL receptor-related proteins 3, 9, 12 (LRP3, 9, 12), and *β*-site amyloid cleavage enzyme 1 (BACE1) (Boucher et al., 2008; Canuel, Libin & Morales, 2009; Doray et al., 2002; Li, Lavigne & Lavoie, 2015; Schmidt et al., 2007; Toh et al., 2018; Zhang et al., 2009). Interestingly, *Drosophila* GGA is related to spermatogenesis. GGA is mainly expressed in the head of female flies, however, in male flies, GGA is not only expressed at high levels in the head but there is also a gender-specific increased expression which is due to the abundant expression of GGA in the testes. Moreover, GGA is expressed at different stages of sperm development, especially in the early stage (Hirst & Carmichael, 2011).

GGA1 is an important member of the GGAs family, located on mouse chromosome 15 and human chromosome 22, and is highly conserved from yeast to mammals (Govero et al., 2012). The current research on GGA1 focuses on its role in the secretion of amyloid beta-peptide (A*β*) and the development of Alzheimer’s disease (AD) (Wahle et al., 2006). In addition, GGA1 also modulates the myogenesis of C2C12 myoblasts (Isobe et al., 2018).

The Golgi apparatus is involved in acrosome formation, and critical for trafficking in spermatogenesis. Moreover, GGA has been shown to be important for spermatogenesis in fruit flies, so we hypothesised that GGA1 might regulate mammalian spermatogenesis. To explore the role of *Gga1* in spermatogenesis, we constructed *Gga1* knockout mice using CRISPR/Cas9 technology. *Gga1* null mice were viable and fully fertile without any obvious abnormalities. However, *Gga1* knockout mice became infertile as a result of poor sperm quality and severely disrupted spermatogenesis when exposed to BPA. In summary, *Gga1* mainly ensures proper spermatogenesis in mice in response to BPA.

## Materials & Methods

### Animal experiments

The *Gga1*-deficient mice (C57BL/6J) were a gift from Hongbin Liu (Shandong University, Jinan, China) and Wei Li (Guangzhou Medical University, Guangzhou, China). The mice were maintained under 12:12 (12-h:12-h light-dark) with a controlled temperature of 23  ± 2 °C. Animals were housed 3-5 mice per cage with free access to food and water. In the first experiment, we used 10 wildtype male mice and 10 knockout male mice to explore the effect of the *Gga1* on the fertility of male mice. In the next experiment, we used six wildtype male mice and six knockout male mice to explore the effect of the *Gga1* on the fertility of male mice under stress. Mice used were randomly separated into control and experimental group. Fourty four mice were used in these experiments. All mice used in the experiments were male, 2-month-old and weighed around 25 g. Two hundred micrograms BPA (239658; Sigma-Aldrich, St. Louis, MO, USA) was added into 10 mL of corn oil and then vortexed until BPA was completely dissolved, and the final concentration was 20 µg/mL. The mixture was stored at 4 °C. Mice in the BPA group were intraperitoneally injected with 40 µg/kg BPA at 5:00 p.m. daily for 2 weeks. Control group received only corn oil by intraperitoneal injection. A 40 µg/kg dose is the same for all animals, and injection volume depends on body weight. For example, a 25 g mouse needs to be injected with a dose of 1 µg/day BPA (volume: 50 µL). The BPA amount used in this study was below the US Environmental Protection Agency (EPA) safe dose of 50 µg/kg/day (Leranth et al., 2008). The primers used for genotyping were primer F1: CCACAGATACTCACCTGACACACGAG and primer R1: CATCTGATGACTGTGGGCAAAGGAG for the mutant allele (604 base pairs) and primer F2: CAGAGATGCTCTGTTTGCGCCA and primer R1: CATCTGATGACTGTGGGCAAAGGAG for the WT allele (840 base pairs). All mice were sacrificed by cervical dislocation, and then immediately dissected to seperate testicles and epididymis. For different experiments, samples were treated in different ways. Testis and epididymis were fixed in 4% PFA for histological analysis or frozen in liquid nitrogen immediately and kept at −80 °C for protein analysis. Only one epididymis per mouse was used to measure sperm motility. All the experimental procedures were carried out in accordance with the guidelines and protocols approved by the Institutional Animal Care and Use Committee (IACUC) protocols of the Institute of Zoology (IOZ-IACUC-2022-252), Chinese Academy of Sciences.

### Immunoblotting

As previously reported, the tunica albuginea of the testis was peeled and seminifeuors tubules and interstitium were homogenized in mortar, supplemented with 1 mmol/L phenylmethylsulphonyl fluoride (PMSF, 0754; Amresco, Solon, OH, USA) and protease inhibitor cocktail (Roche, 04693116001; Roche Diagnostics, Rotkreuz, Switzerland) (Zhang et al., 2022). After homogenization and transient sonication, the samples were lysed on ice for 30 min. Then the samples were centrifuged at 12,000 rpm for 20 min at 4 °C. Next, the supernatants were transferred to new tubes. The total protein concentrations were determined using a BCA assay (Boster, Wuhan, China). Approximately 20 µg of total protein was separated by 10% SDS-PAGE, and then transferred to the nitrocellulose membranes. Afterward, membranes were blocked in 5% skimmed milk (DifcoTM Skim Milk, BD) at room temperature for 1 h. After washing the membranes with 1XPBS 3 times for 5 minutes each time, then, the membranes were incubated with primary antibody at 4 °C overnight and secondary antibody for 1 h at room temperature. Primary antibodies used were anti-GGA1 (Santa Cruz Biotechnology, Santa Cruz, CA, USA, diluted 1:200), anti-Tubulin (ABclonal, Woburn, MA, USA, diluted 1:500). The secondary antibodies used were IRDye® 680CW Goat anti-Mouse (1:10000; LI-COR Biosciences, Lincoln, NE, USA) and IRDye® 800CW Goat anti-Rabbit (1:10000; LI-COR Biosciences, Lincoln, NE, USA). Next, the membranes were washed 3 times with 1XPBS, and incubated with the secondary antibodies at room temperature for 1 h. Finally, the membranes were scanned using the ODYSSEY CLx Infrared Imaging System (LI-COR Biosciences, Lincoln, NE, USA).

### Epididymal sperm motility and sperm count assay

Cauda epididymal sperm were released into 1ml 1X PBS and incubated for 10 min at 37 °C. The swim-up suspension was used for sperm motility analysis using a custom-made animal semen analysis system from SAS MEDICAL (Cary, NC, USA). The samples were analyzed *via* computer-assisted semen analysis (CASA) software developed by SAS MEDICAL (Cary, NC, USA). Sperm count was obtained using a hemocytometer.

### Fertility assay

The male fertility tests were performed as previously described (Chen et al., 2021). A 2-month-old male mouse was crossed with two 8-week-old wild-type female mice. Female mice were separated and checked for copulatory plugs after mating. The plugged females were separated into individual cages for monitoring pregnancy. If a female generated no pups by day 22 postcoitus, the mice were deemed not pregnant.

### Tissue collection and histological analysis

Testes and cauda epididymides were fixed in Bouin’s fixative (41506; Sigma, Waltham, MA, USA) for 24 h. Next, the tissues were dehydrated in graded ethanol and embedded in paraffin. Five-micrometer sections were cut and mounted on glass slides, then stained with hematoxylin and eosin (H&E) or periodic acid schiff (PAS). Finally, images were obtained using a Nikon Eclipse TS100 inverted microscope (Nikon, Tokyo, Japan).

### Immunofluorescence and TUNEL assay

The cauda epididymal sperm samples were spread onto the surface of the slides, and then dried naturally overnight at room temperature. These slides were fixed in 4% PFA for 10 min, and permeabilized with 0.5% Triton X-100 for 10 min, and blocked in 5% bovine serum albumin (BSA, AP0027, Amresco, Boise, ID, USA) in PBS for 30 min at room temperature. Then, the slides were incubated with the fluorescent lectin peanut agglutinin (PNA, L21409, 1:400; Thermo Fisher Scientific, Waltham, MA, USA) to analyze the status of the acrosome. The cell death was measured using terminal deoxynucleotidyl transferase dUTP nick end-labeling (TUNEL) assay kit (In Situ Cell Death Detection Kit, 11684817910, Roche, La Posay, France), according to the manufacturer’s protocol. The nuclei were stained with 4′, 6-diamidino-2-phenylindole (DAPI, D3571, Thermo Fisher Scientific, Waltham, MA, USA) for 5 min. Finally, the images were taken by a Zeiss LSM 880 microscope.

### Statistical analysis

All data were presented as the mean  ± SD. Unpaired t-tests and two-way ANOVA with post hoc tests were used for statistical analysis. Differences were considered significant at *P* < 0.05 (*), *P* < 0.01 (**), *P* < 0.001 (***), and *P* < 0.0001 (****). NS stands for not statistically significant.

## Results

### *Gga1*^−/−^ male mice were successfully generated and displayed normal spermatogenesis

**Figure 1:**
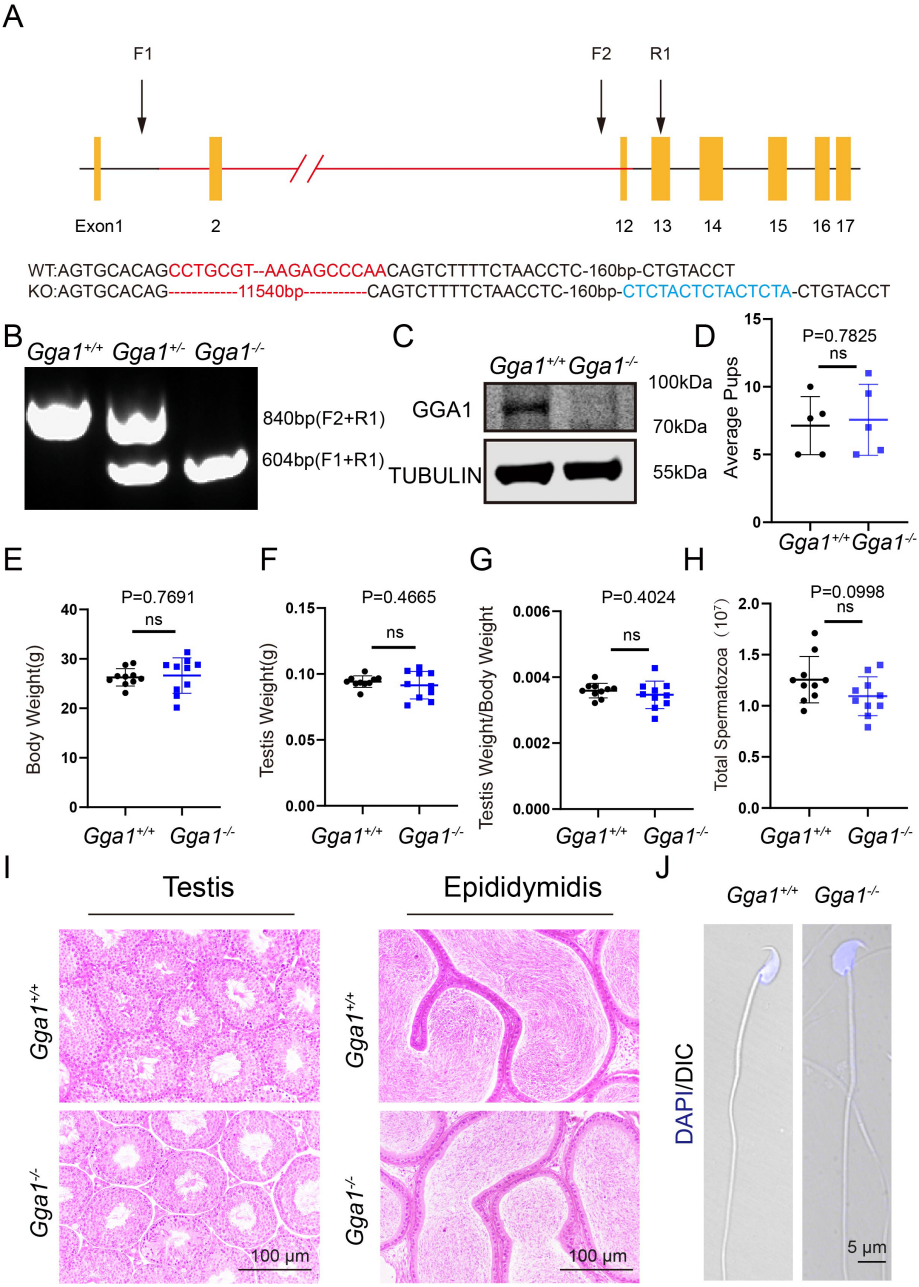
*Gga1*^-/-^ mice were generated and exhibited no obvious reproductive abnormality. (A) Schematic strategy of *Gga1*^-/-^ mice construction by CRISPR-Cas9-mediated genome editing. Sanger sequencing results of knockout mice confirmed the successful deletion of 11,540 bp and a 15 bp insert in *Gga1* gene. (B) Genotyping of *Gga1*^+/+^, *Gga1*^+/-^ and *Gga1*^-/-^ mice. (C) The GGA1 protein was completely absent in the testis of *Gga1*^-/-^ mice. Immunoblotting of GGA1 was performed in *Gga1*^+/+^ and *Gga1*^-/-^ testis. TUBULIN served as a loading control. (D) The fertility assessment experiments were performed in *Gga1*^+/+^ and *Gga1*^-/-^ male mice, *n* = 5. (E) Body weight of 2-month-old *Gga1*^+/+^ and *Gga1*^-/-^ male mice, *n* = 10. (F) Testis weight of 2-month-old *Gga1*^+/+^ and *Gga1*^-/-^ male mice, *n* = 10. (G) The ratios of testes weight to body weight for 2-month-old *Gga1*^+/+^ and *Gga1*^-/-^ male mice, *n* = 10. (H) The sperm counts in the caudal epididymides of 2-month-old *Gga1*^+/+^ and *Gga1*^-/-^ mice, *n* = 10. (I) Histological analysis of the testes and caudal epididymidis of *Gga1*^+/+^ and *Gga1*^-/-^ male mice by H&E staining. (J) Immunofluorescence staining of DAPI (blue) in *Gga1*^+/+^ and *Gga1*^-/-^ spermatozoa.

To investigate the function of *Gga1* in spermatogenesis, we generated *Gga1*^−/−^ mice using CRISPR/Cas9 technology (Fig. 1A). Sanger sequencing and genotyping confirmed that 11,540 bp was deleted and an unexpected additional 15 bp was inserted in *Gga1* gene (Figs. 1A and 1B). The immunblotting analysis showed GGA1 protein was completely absent in *Gga1*^−/−^ testis compared with *Gga1*^+/+^ testis (Fig. 1C). These results demenstrated that we had sucessfully generated *Gga1* knockout mice.

To determine whether loss of GGA1 affected reproductive system, we next examined the fertility of 2-month-old *Gga1*^−/−^ and *Gga1*^+/+^ male mice and found no significant difference in average pups between the two groups (Fig. 1D). Other than that, *Gga1*^−/−^ mice were viable and displayed no obvious differences in development or behavior compared with *Gga1*^+/+^ mice. The body weight, absolute testis weight, and relative testis weight of male mice were examined, and no significant differences were observed between the two groups (Figs. 1E–1G).

To examine more subtle reproductive damages, the total number of spermatozoa in the cauda epididymis was counted. The data showed that *Gga1*^−/−^ male mice had normal sperm numbers (Fig. 1H). Besides, the histological analysis revealed that the spermatogenic cells in the seminiferous epithelium of *Gga1*^−/−^ male mice were tightly arranged, and no obvious abnormalities were observed in spermatogenesis. In addition, sperm density of *Gga1*^−/−^ mice also appeared to be normal compared with those of wild-type littermates (Fig. 1I). Sperm morphology assessment was also an important criterion for evaluating sperm quality. Therefore, single-sperm immunofluorescence staining was performed and imaged using confocal microscopy. Finally, our results indicated that the sperm of *Gga1*^−/−^ male mice had normal morphology compared with *Gga1*^+/+^ male mice (Fig. 1J). Taken together, we concluded that knockout of *Gga1* had no obvious effect on development and reproduction in male mice under normal condition.

### *Gga1*^−/−^ male mice became infertile after BPA exposure

**Figure 2:**
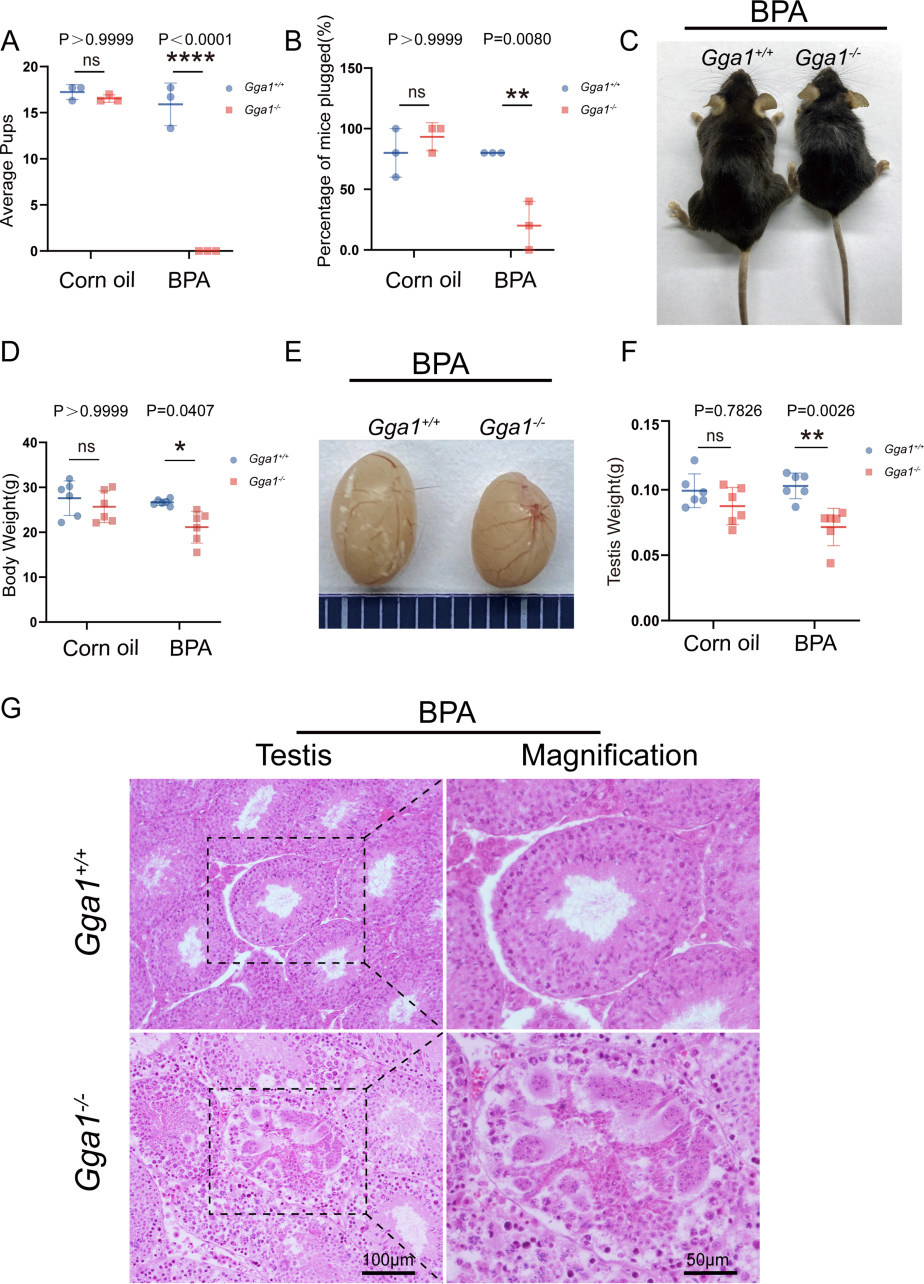
*Gga1*^-/-^ male mice were sterile after intraperitoneal injection of BPA. (A) The fertility assessment experiment was performed in BPA-treated *Gga1*^+/+^ and *Gga1*^-/-^ male mice. BPA-treated *Gga1*^-/-^ male mice produced no offspring, *n* = 3. (B) *Gga1*^-/-^ male mice showed reduced mating ability, *n* = 3. (C) The picture of BPA-treated *Gga1*^+/+^ and *Gga1*^-/-^ male mice, *n* = 6. (D) The body weight of BPA-treated *Gga1*^-/-^ male mice was significantly reduced compared to that of control littermates. (E) The testis picture of BPA-treated *Gga1*^+/+^ and *Gga1*^-/-^ male mice. (F) After BPA exposure, the testes weight of *Gga1*^-/-^ mice decreased significantly compared to that of control littermates, *n* = 6. (G) Histological analysis of testes in BPA-treated *Gga1*^+/+^ and *Gga1*^-/-^ male mice by H&E staining. Testis sections of control mice demonstrated normal cell populations within the seminiferous epithelium, whereas spermatogenic cells showed severe defects within the disrupted seminiferous tubules of BPA-treated *Gga1*^-/-^ male mice.

It has been defined that *Gga1* is located in Golgi, which is essential for acrosome formation, we speculated that *Gga1*^−/−^ mice might be more sensitive to the environmental pollutants that affect the Golgi apparatus. In order to demonstrate the role of *Gga1* in spermatogenesis under stressful conditions, we treated the 2-month-old *Gga1*^+/+^ and *Gga1*^−/−^ male mice with a low dose of BPA (40 µg/kg/day) for two weeks. Then we performed fertility tests, and found that BPA-treated *Gga1*^+/+^ male mice were still fertile, while BPA-treated *Gga1*^−/−^ male mice were sterile (Fig. 2A). To assess mating behavior of male mice, we examined their ability to form copulatory plugs after mating. The results showed that BPA-treated *Gga1*^−/−^ male mice exhibited abnormal mounting behaviors and the percentage of plugged females was decreased significantly compared with that of wild-type mice (Fig. 2B). Besides that, BPA-treated *Gga1*^−/−^ male mice showed smaller body size and lower body weight compared with those of BPA-treated control males (Fig. 2C and 2D). Anatomical inspection revealed that the testes of BPA-treated *Gga1*^−/−^ male mice were significantly reduced in size and weight compared to those of BPA-treated *Gga1*^+/+^ male mice (Fig. 2E and 2F). To discover the cause of male infertility and reduced testis size, furtherly, we examined the testes of *Gga1*^+/+^ and *Gga1*^−/−^ mice by H&E staining. The histological analysis showed that *Gga1*^+/+^ male mice exhibited normal spermatogenesis with a regular arrangement of the spermatogenic epithelium in the seminiferous tubules after BPA treatment. However, *Gga1*^−/−^ mice displayed various testicular injuries, including disordered tubular structures, aggregation of spermatogenic cells and vacuoles in seminiferous tubules. Profoundly, nuclei near the basement membrane were abnormal shaped, enlarged, and extremely dark staining (Fig. 2G). In summary, the testes of *Gga1*^−/−^ male mice exposed to BPA were damaged, along with reduced body size.

### BPA-treated *Gga1*^−/−^ male mice showed decreased sperm motility and quantity and increased sperm malformations

**Figure 3:**
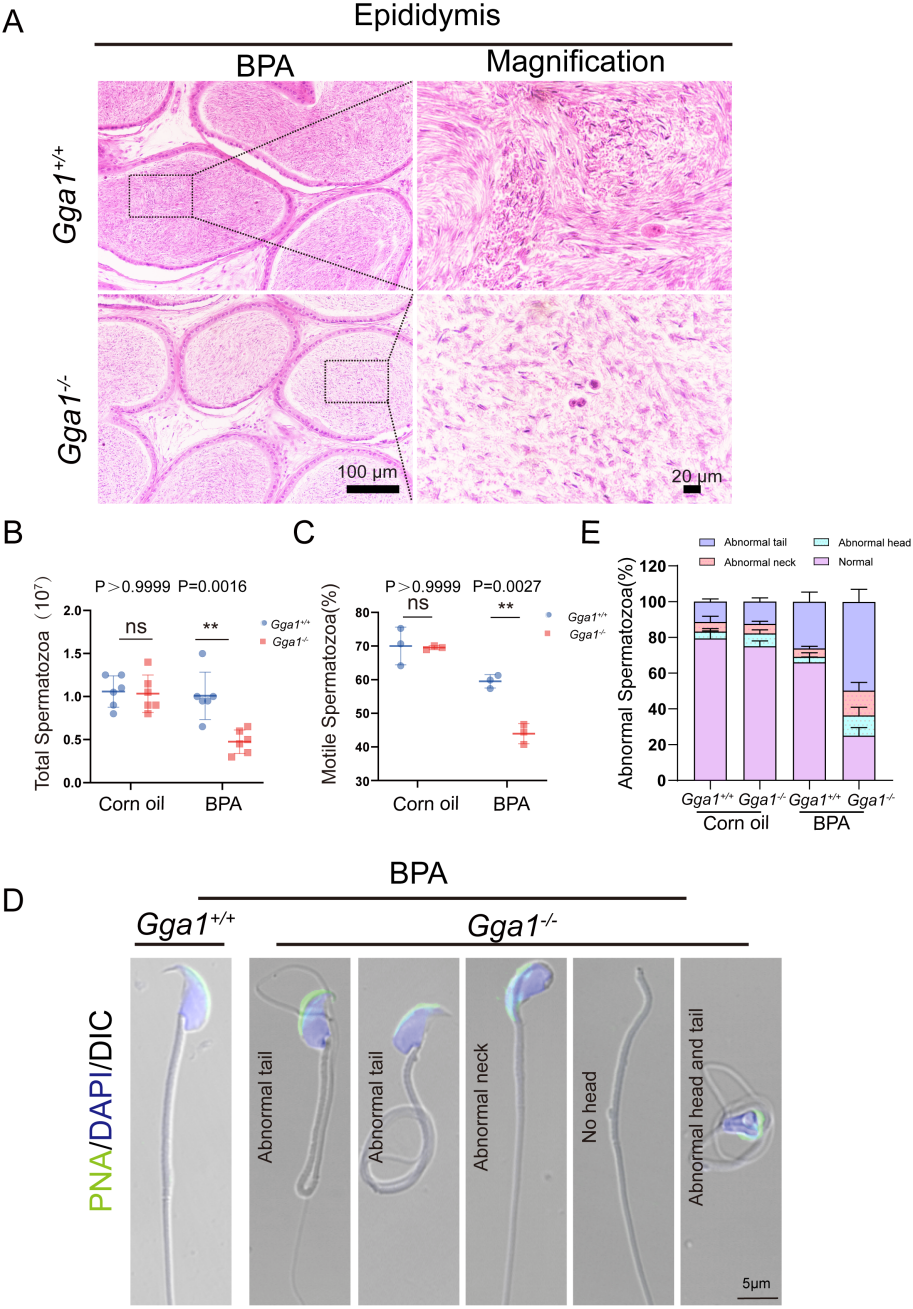
Sperm quality was significantly reduced in BPA-exposed *Gga1*^-/-^ male mice. (A) Histological analysis of the caudal epididymides of BPA-treated *Gga1*^+/+^ and BPA-treated *Gga1*^-/-^ male mice by H&E staining. (B) The sperm count of BPA-exposed *Gga1*^-/-^ male mice were decreased, *n* = 6. (C) The sperm motility of BPA-exposed *Gga1*^-/-^ male mice were decreased, *n* = 3. (D) Single-sperm immunofluorescence analysis with the acrosome-specific marker PNA (green) was performed using *Gga1*^+/+^ and *Gga1*^-/-^ spermatozoa treated with BPA. Nuclei were stained with DAPI (blue). (E) The percentage of different abnormal spermatozoa in cauda epididymis of *Gga1*^+/+^ and *Gga1* ^-/-^ male mice when treated with corn oil and BPA, *n* = 3.

Adequate sperm count, vigorous sperm motility, and perfect sperm morphology are key factors in normal male reproduction, and defects in these factors often lead to infertility. To explore the reason of the infertility of BPA-treated *Gga1*^−/−^ male mice, we performed a histological analysis of the caudal epididymis by H&E staining and found fewer spermatozoa in the epididymal lumen of BPA-treated *Gga1*^−/−^ mice than that of BPA-treated *Gga1*^+/+^ mice (Fig. 3A). We next released spermatozoa from the epididymis and found that sperm numbers from BPA-treated *Gga1*^−/−^ mice were significantly lower than those of BPA-treated *Gga1*^+/+^ mice (Fig. 3B). In addition, the percentage of motile spermatozoa decreased sharply in BPA-treated *Gga1*^−/−^ mice compared with that of BPA-treated *Gga1*^+/+^ mice (Fig. 3C). To further examine the morphology of mature spermatozoa from the cauda epididymis, we used PNA to assess the sperm acrosomal status, and nuclei were costained with DAPI. The results showed that only few sperm deformities were detected in the BPA-treated *Gga1*^+/+^ mice, however, BPA-treated *Gga1*^−/−^ mice showed a high rate of malformed spermatozoa, including abnormal tail or neck, no head, and abnormal head and tail. Normal sperm head with bent neck or different curly tail and abnormal sperm head with normal or curly tail were the major defect categories among abnormalities in the *Gga1*^−/−^ sperms. These results demenstrated that BPA exposure jeopardized sperm morphology of *Gga1*^−/−^ males compared with the control group (Fig. 3D). The percentage of spermatozoa with abnormal tail, neck or head were shown in (Fig. 3E). These data confirmed that BPA exposure reduced sperm quality of *Gga1*^−/−^ male mice.

### Effect of BPA on spermatogenesis of *Gga1*^−/−^ mice

**Figure 4:**
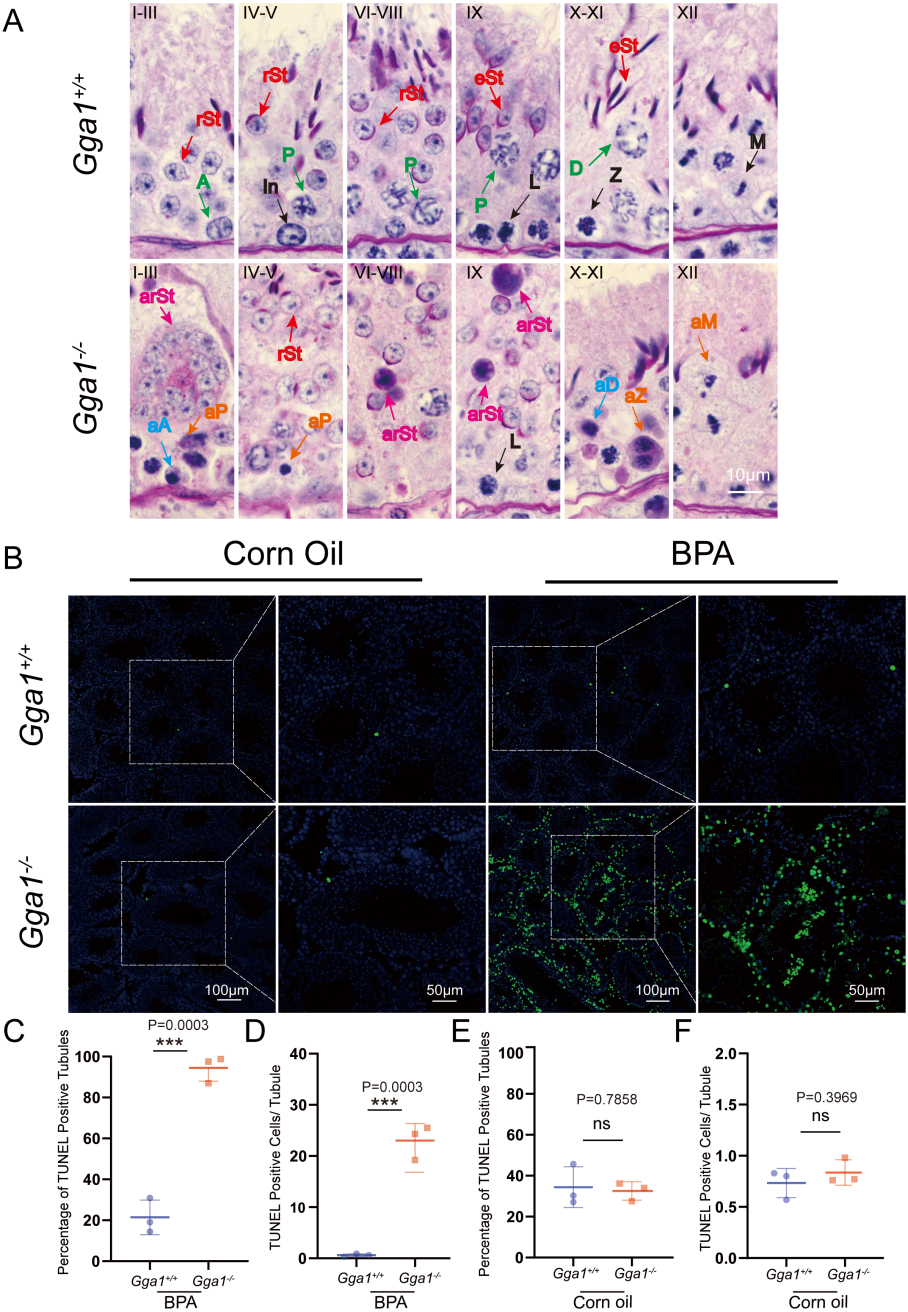
BPA treatment induced abnormal spermatogenesis and increased cell death in testes lacking *Gga1*. (A) Paraffin sections of seminiferous tubules from BPA-treated *Gga1*^+/+^ and *Gga1*^-/-^ males were stained with PAS-hematoxylin. A, type A spermatogonia; In, intermediate spermatogonia; L, leptotene spermatocytes; Z, zygotene spermatocytes; P, pachytene spermatocytes; D, diplotene spermatocytes; M, meiotic divisions; rSt, round spermatids; eSt, elongating spermatids; aA, abnormal type A spermatogonia; aD, abnormal diplotene spermatocytes; aP, abnormal pachytene spermatocytes; aZ, abnormal zygotene spermatocytes; arSt, abnormal round spermatids, aM abnormal meiotic divisions. (B) TUNEL staining was performed in testes sections from corn oil or BPA-exposed *Gga1*^+/+^ and *Gga1*^-/-^ males. (C) The percentage of TUNEL positive tubules in BPA-injected male mice, *n* = 3. (D) The average number of TUNEL positive cells per tubule in BPA-injected male mice, *n* = 3 . (E) The percentage of TUNEL positive tubules in corn oil-injected male mice, *n* = 3. (F) The average number of TUNEL positive cells per tubule in corn oil-injected male mice, *n* = 3.

To evaluate BPA-induced testicular damage in *Gga1*^−/−^ mice, we thoroughly investigated the spermatogenesis in BPA-treated *Gga1*^+/+^ and *Gga1*^−/−^ mice by PAS-hematoxylin staining (Ahmed & De Rooij, 2009) and roman numerals indicated the spermatogenic stages of the seminiferous tubule (Fig. 4A). Histological analysis revealed that the seminiferous tubules of BPA-treated *Gga1*^+/+^ males remained well-structured and arranged in an orderly manner. However, BPA-treated *Gga1*^−/−^ males exhibited a complete disruption of spermatogenesis, including disorganized seminiferous epithelium, degenerative cell structure ranging from cellular breakdown to nuclear condensation, abnormal formation of syncytial multinucleated giant cells and increased cell death in the cycle of the seminiferous epithelium (Fig. 4A).

BPA has been reported to induce cell death in multiple studies (Guo et al., 2017; Lee et al., 2007; Urriola-Muñoz, Lagos-Cabré & Moreno, 2014). Then, we performed TUNEL assays and found that the total number of TUNEL-positive cells in testes, the percentage of TUNEL positive tubules and the average number of TUNEL positive apoptotic cells per tubule from BPA-treated *Gga1*^−/−^ males were significantly increased compared with those of BPA-treated control mice (Figs. 4B, 4C and 4D). However, there was no significant difference observed in TUNEL staining between testes from *Gga1*^+/+^ males and *Gga1*^−/−^ males in the corn oil group (Figs. 4E and 4F). In conclusion, our results suggested that loss of *Gga1* in male mice combined with BPA exposure led to considerable spermatogenic disruption with plenty of germ cell death. Thus, GGA1 may ensure normal spermatogenesis in response to harmful conditions.

## Discussion

The *Gga1* gene was highly expressed in *Drosophila* testis and related to spermatogenesis (Hirst & Carmichael, 2011). However, the role of *Gga1* in mammalian spermiogenesis has not been well investigated. In this study, we explored the function of *Gga1* in spermatogenesis by creating *Gga1* knockout (KO) mice. These KO mice manifested dramatically damaged spermatogenesis and were infertile when exposed to BPA, suggesting an essential role of *Gga1* in spermatogenesis under stress.

Govero et al. (2012) previously reported that mice homozygous null for *Gga1* alleles exhibited normal fertility and gained weight somewhat slower than their WT littermates. In this study, we also found *Gga1*^−/−^ male mice were fertile under normal condition (Fig. 1D), which was consistent with the previously reported data. On the contrary, our data showed that the knockout of *Gga1* had no obvious effect on the weight of the male mice(Fig. 1E), which was inconsistent with the published data. This difference may be due to different number of mice used. GGA was a highly expressed gene in the testes of fruit flies, even if its expression was reduced to below 5%, spermatogenesis is still normal (Hirst & Carmichael, 2011). Our study showed that male mice remain fertile after knocking out *Gga1* alone. In conclusion, the function of the *GGA* gene was conserved between mice and fruit flies.

Several genes, such as *Mk2* and *Ggnbp1* had no obvious effect on spermatogenesis under normal circumstances, but can protect the normal progress of spermatogenesis under stress (Han et al., 2020; Williams et al., 2019; Williams et al., 2016). Under normal conditions, the knockout of *Gga1* exerted no effect on the fertility of male mice. Once the mice were treated with BPA for 14 days, the *Gga1*^−/−^ males were sterile and showed impaired male reproductive system. These results were in accordance with the BPA toxicity on the reproductive system in previous studies. BPA has been known to damage male germ cell proliferation, reduce sperm count and motility, increase the number of abnormal sperm and cause DNA damage (Liu, Wang & Liu, 2021; Qiu et al., 2013). Here, our results confirmed that *Gga1* can ensure normal spermatogenesis under BPA treatment.

The Golgi apparatus is responsible for acrosome formation during spermatogenesis (Khawar, Gao & Li, 2019). In the early stages of spermatid development, the Golgi-derived small vesicles gather in the concave area of the sperm nucleus, and along with sperm development, the small vesicles fuse to form large acrosome granules (Venditti & Minucci, 2022). *PICK1* and *Gopc* gene are essential for acrosome formation in male germ cells (Bizkarguenaga et al., 2019; Du et al., 2023). PICK1, the protein interacting with C kinase 1, plays an important role in Golgi-derived vesicle trafficking, and its deficiency in the male reproductive system results in abnormal vesicle trafficking from Golgi to acrosome, which eventually disrupts acrosome formation and leads to male infertility (Du et al., 2023). *Gga1* and PICK1 have the same subcellular localization, we guessed that the functions of the two genes would be very similar (Binkle et al., 2022; Zhang et al., 2019). So, like PICK1, *Gga1* may be involved in the process of spermatogenesis in mice by participating in the transport of Golgi vesicles. It has been reported that BPA exposure affects the structure of the Golgi apparatus by significantly decreasing Golgi proteins, such as GM130 (Pan et al., 2021). The Golgi of *Gga1*^−/−^ mice was more likely to be affected by BPA treatment. Under normal conditions, the normal spermatogenesis of *Gga1*^−/−^ male mice may be due to redundancy with other members of the GGAs family. However, the role of GGA2 and GGA3 in the process of spermatogenesis are still unknown. Thus, we can detect changes in the expression of GGA2 and GGA3 in *Gga1*^−/−^ null mice, then their potential functions can be revealed. Perhaps we can knockout other genes in GGAs family to explore the function of *Gga1*.

However, our study has some limitations. The underlying mechanism of BPA treatment causes defects in spermatogenesis of *Gga1*^−/−^ mice needs to be further elucidated. Whether the microstructure of the head, neck, and tail of sperm is damaged requires an in-depth and detailed study. The relationship between GGA1 and acrosomes should be further clarified. Whether *Gga1*^−/−^ female mice are infertile in response to BPA treatment also need to be further explored. Further studies would allow us to discover whether other environmental contaminants affect *Gga1*^−/−^ male mice.

## Conclusions

In summary, our results showed that *Gga1* ablation disturbed spermatogenesis of BPA-treated male mice. *Gga1* may be a potential target for therapy of male infertility caused by environmental pollutants.

##  Supplemental Information

10.7717/peerj.15673/supp-1 Supplemental Information 1 Raw data for figures Markers show which part of the captured picture was taken.

10.7717/peerj.15673/supp-2 Supplemental Information 2 Author Checklist

10.7717/peerj.15673/supp-3 Supplemental Information 3 The raw data of Figure 1 All raw data in Figure 1.

10.7717/peerj.15673/supp-4 Supplemental Information 4 The raw data of Figure 2

10.7717/peerj.15673/supp-5 Supplemental Information 5 The raw data of Figure 3

10.7717/peerj.15673/supp-6 Supplemental Information 6 The raw data of Figure 4

## Additional Information and Declarations

### Data Availability

The following information was supplied regarding data availability: The raw measurements are available in the Supplemental Files.
