# Supplementary material for: GGA1 participates in spermatogenesis in mice under stress
Source: PeerJ. 2023 Aug 3;11:e15673. doi: 10.7717/peerj.15673 (PMC10404397; doi:10.7717/peerj.15673)
Supplement: Supplemental Information 1 — Markers show which part of the captured picture was taken. [file peerj-11-15673-s001.pdf]

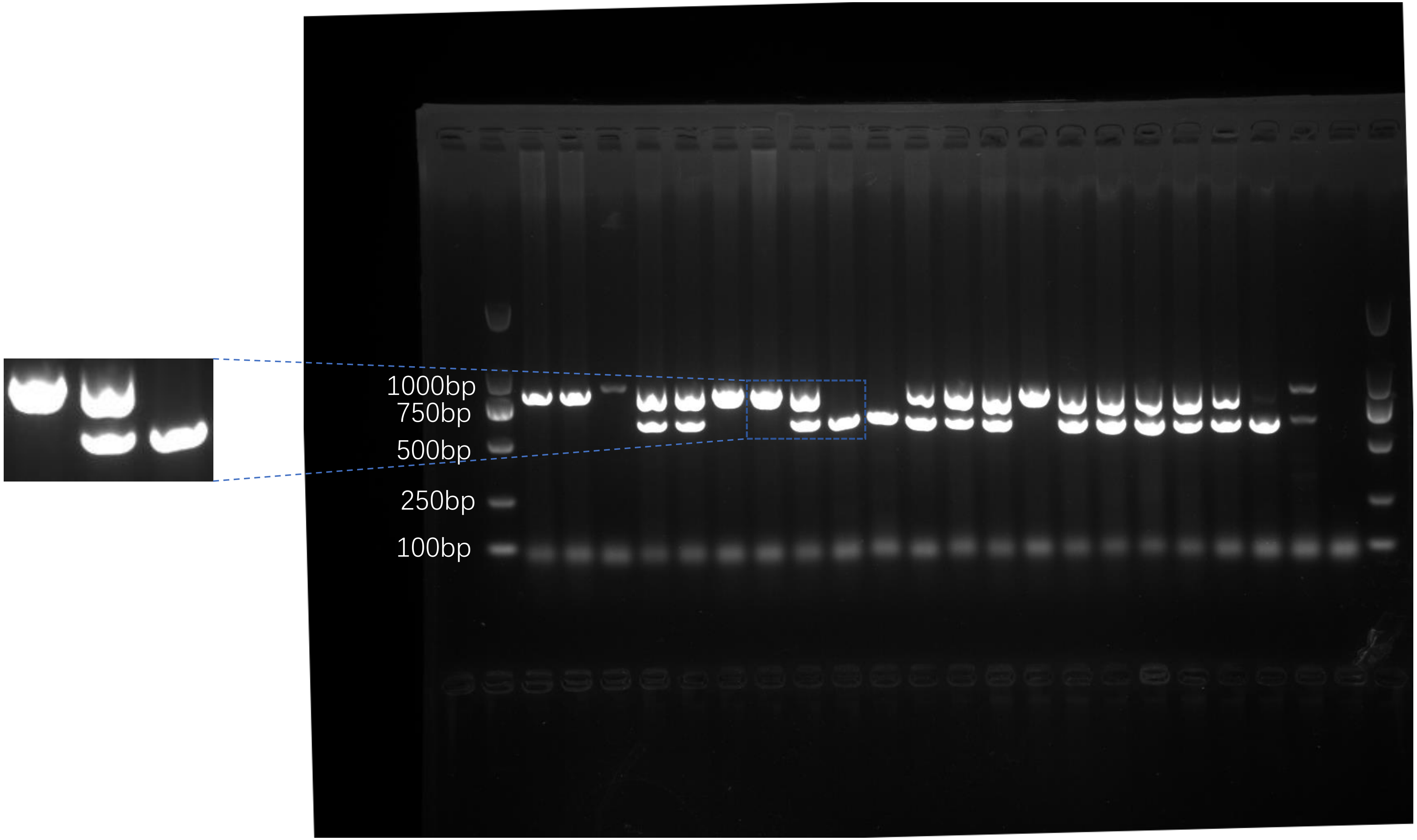

Figure1B: Genotyping of *Gga1+/+*, *Gga1+/−* and *Gga1-/-* mice.

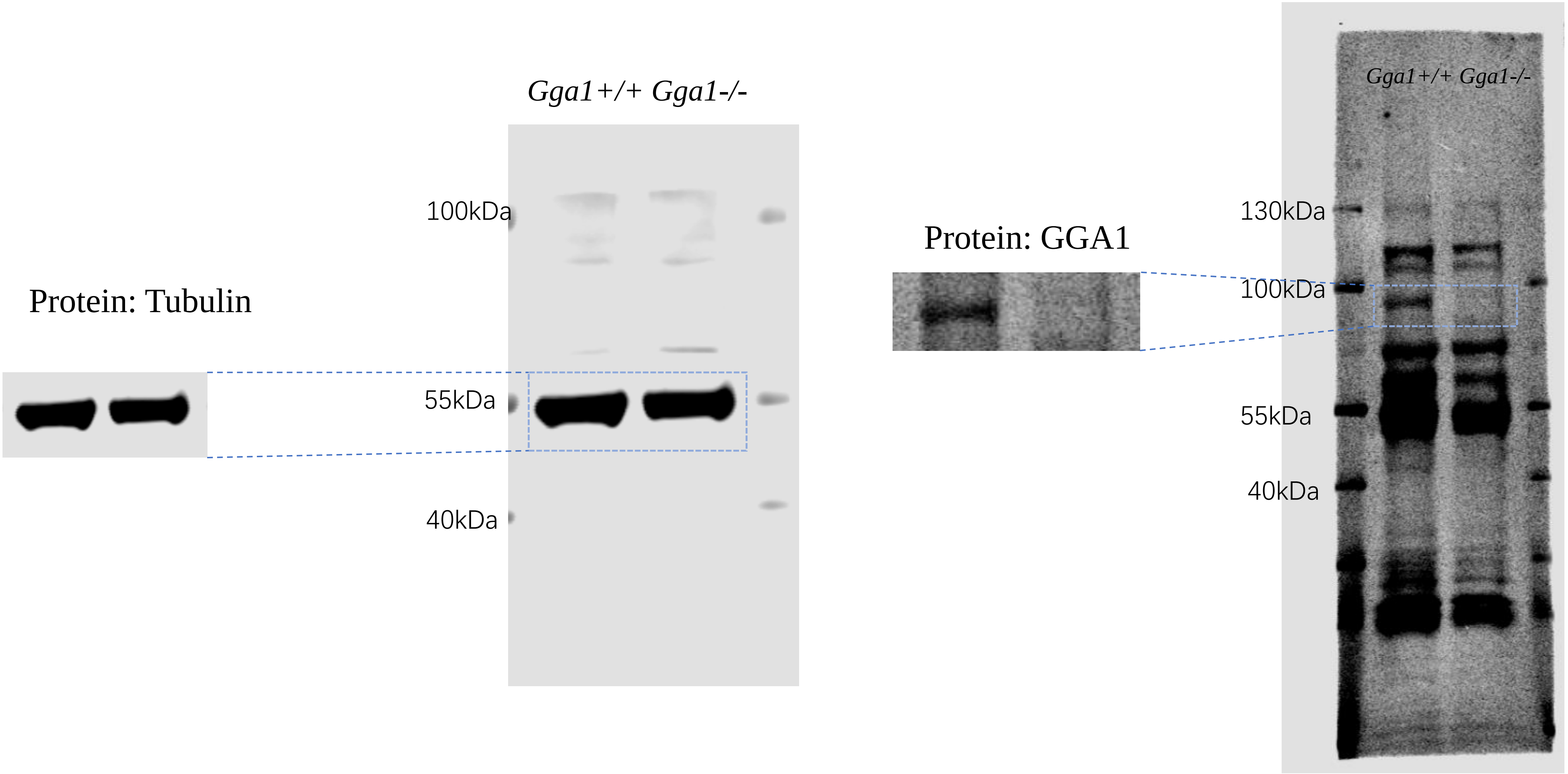

Figure1C: The GGA1 protein was completely absent in the testis of *Gga1-/-* mice. Immunoblotting of GGA1 was performed in *Gga1+/+* and *Gga1-/-* testis. TUBULIN served as a loading control..

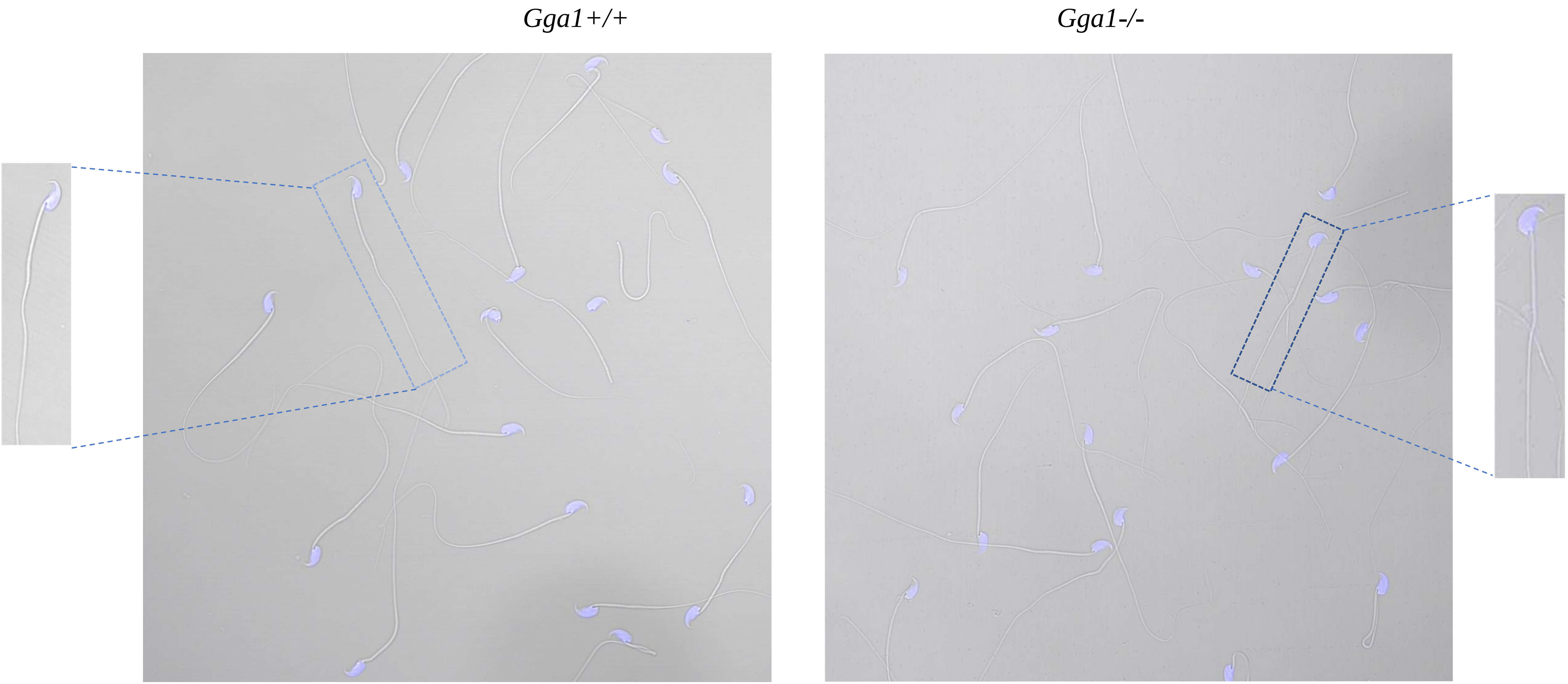

Figure1J: Immunofluorescence staining of DAPI in *Gga1+/+* and *Gga1-/-* spermatozoa.

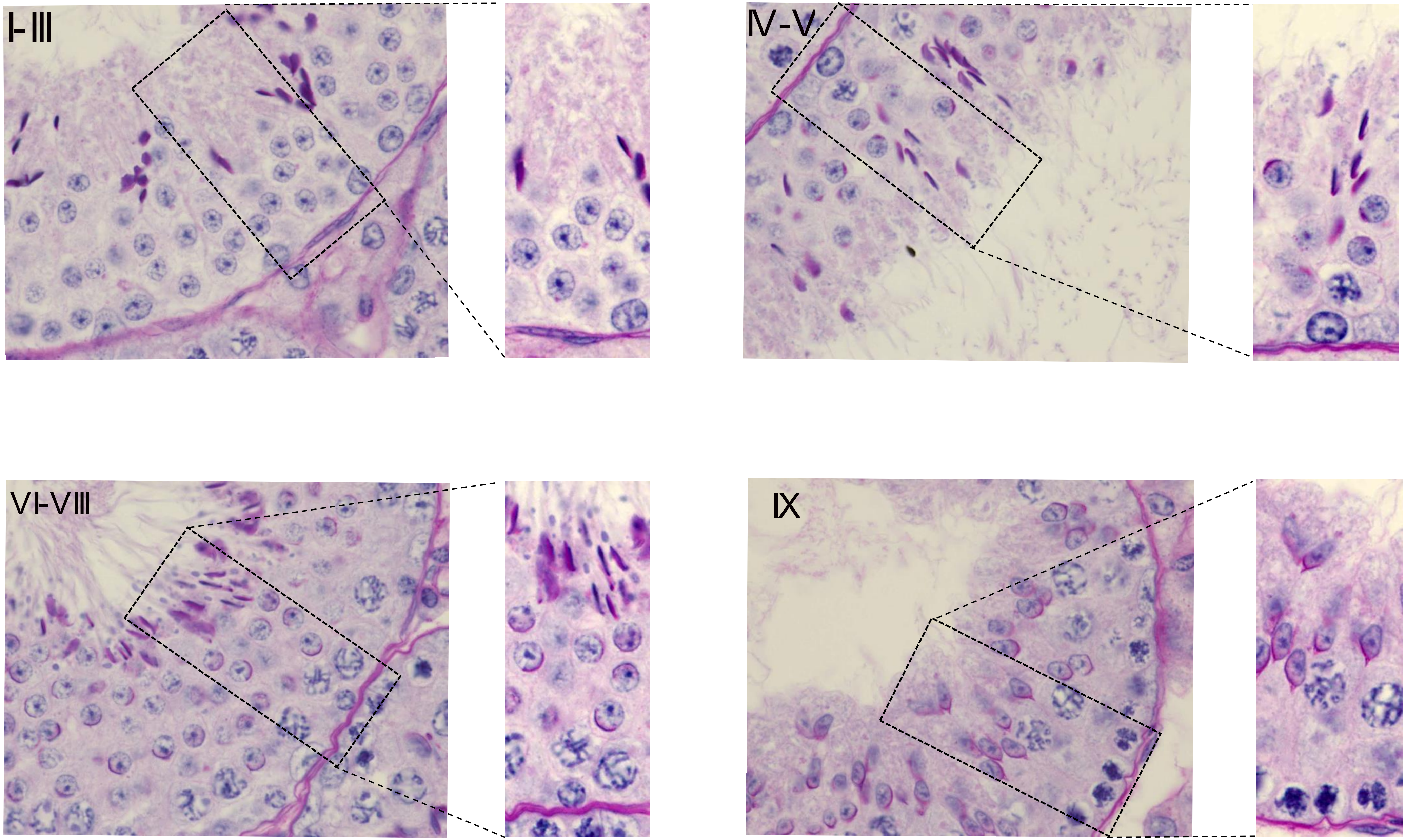

Figure4B: Paraffin sections of seminiferous tubules from BPA-treated *Gga1*$^{+/+}$ males were stained with PAS-hematoxylin.

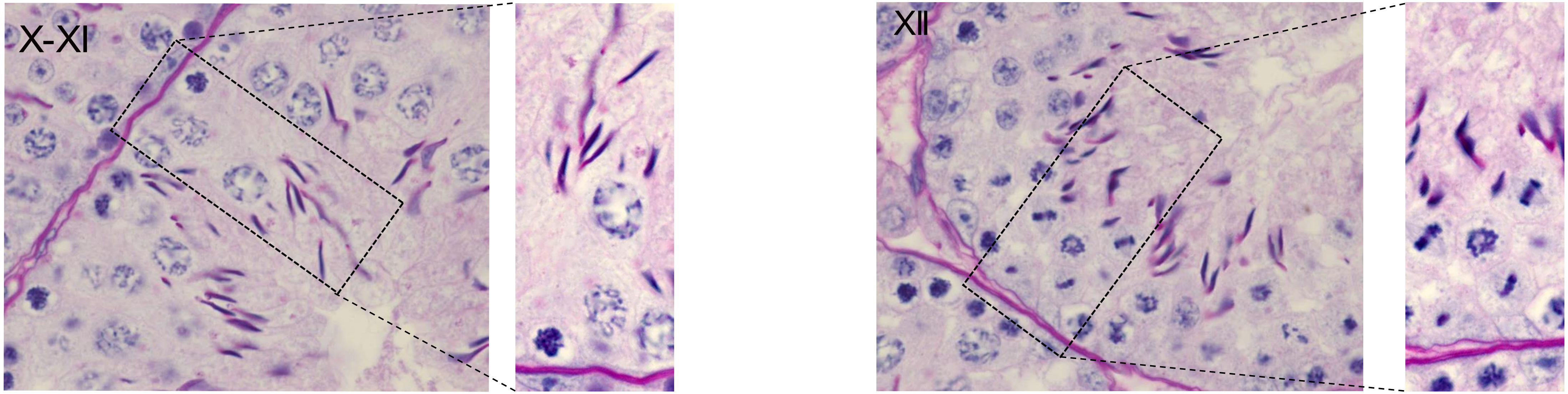

Figure4B: Paraffin sections of seminiferous tubules from BPA-treated *Gga1*$^{+/+}$ males were stained with PAS-hematoxylin.

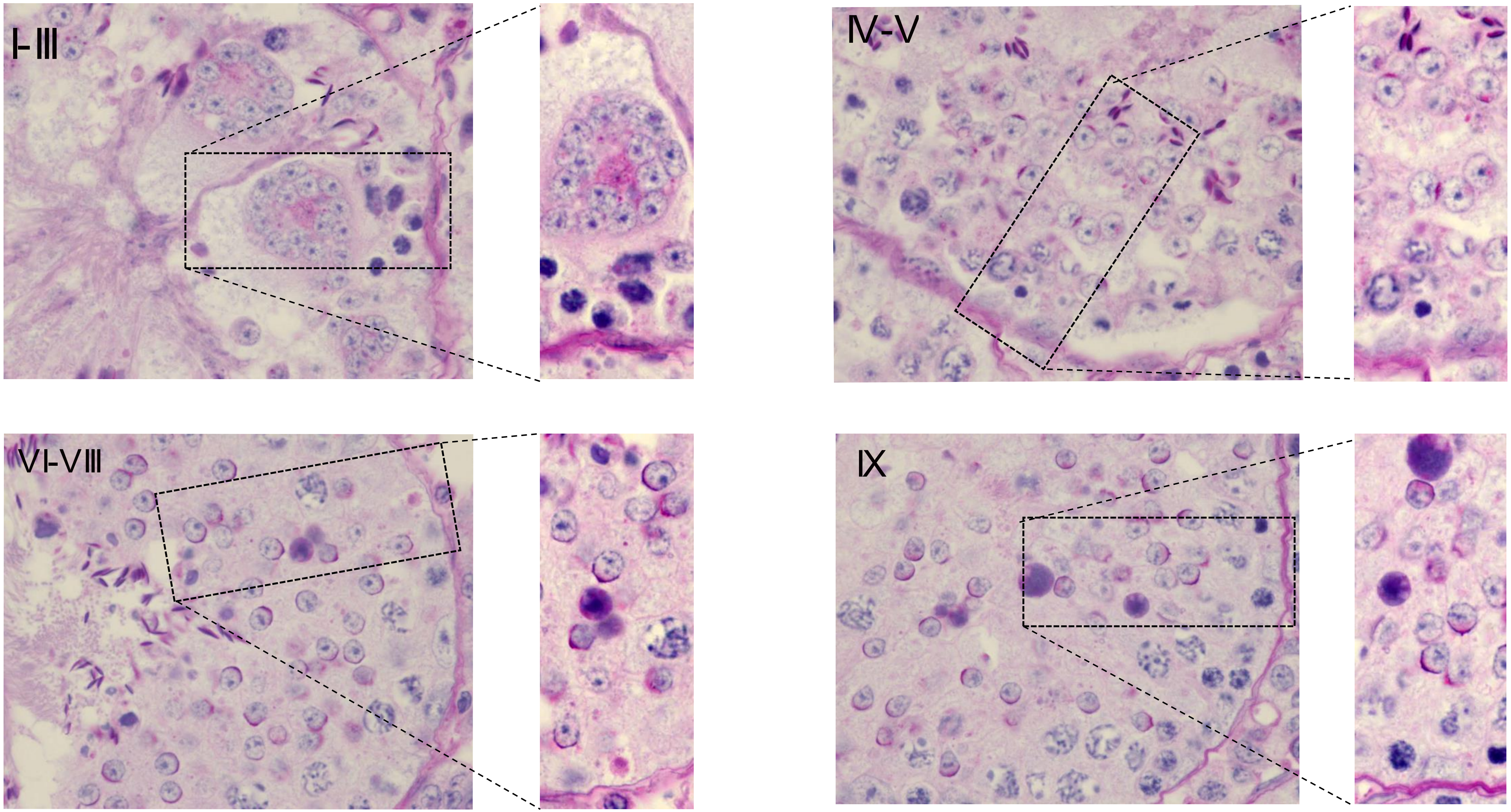

Figure4B: Paraffin sections of seminiferous tubules from BPA-treated *Gga1*$^{-/-}$ males were stained with PAS-hematoxylin.

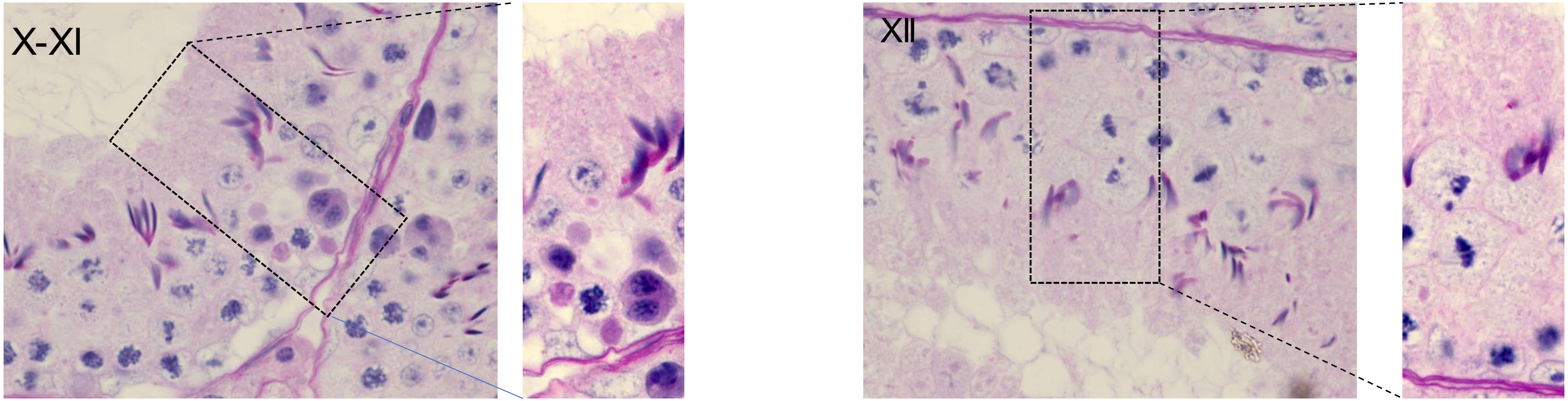

Figure4B: Paraffin sections of seminiferous tubules from BPA-treated *Gga1*$^{-/-}$ males were stained with PAS-hematoxylin.
